# A First Expression, Purification and Characterization of Endo-β-1,3-Glucanase from *Penicillium expansum*

**DOI:** 10.3390/jof9100961

**Published:** 2023-09-25

**Authors:** Kaili Wang, Siyu Huai, Zhuqing Tan, Guillaume Legrand Ngolong Ngea, Esa Abiso Godana, Jun Shi, Qiya Yang, Xiaoyun Zhang, Lina Zhao, Hongyin Zhang

**Affiliations:** School of Food and Biological Engineering, Jiangsu University, Zhenjiang 212013, China; 15981843773@163.com (K.W.); 13645684231@163.com (S.H.); 15851496605@163.com (Z.T.); ngolngea@yahoo.fr (G.L.N.N.); esa.abiso@hotmail.com (E.A.G.); 18365148903@163.com (J.S.); yangqiya1118@163.com (Q.Y.); zhangxiaoyungu@126.com (X.Z.); linabobo0706@163.com (L.Z.)

**Keywords:** endo-β-1,3-glucanase, *Penicillium expansum*, expression purification, enzymatic characteristics

## Abstract

β-1,3-glucanase plays an important role in the biodegradation, reconstruction, and development of β-1,3-glucan. An endo-β-1,3-glucanase which was encoded by *PeBgl1* was expressed, purified and characterized from *Penicillium expansum* for the first time. The *PeBgl1* gene was amplified and transformed into the competent cells of *E. coli* Rosetta strain with the help of the pET-30a cloning vector. The recombinant protein PeBgl1 was expressed successfully at the induction conditions of 0.8 mmol/L IPTG at 16 °C for 16 h and then was purified by nickel ion affinity chromatography. The optimum reaction temperature of PeBgl1 was 55 °C and it had maximal activity at pH 6.0 according to the enzymatic analysis. Na_2_HPO_4_-NaH_2_PO_4_ buffer (pH 6.0) and NaCl have inhibitory and enhancing effects on the enzyme activities, respectively. SDS, TritonX-100 and some metal ions (Mg^2+^, Ca^2+^, Ba^2+^, Cu^2+^, and Zn^2+^) have an inhibitory effect on the enzyme activity. The results showed that PeBgl1 protein has good enzyme activity at 50–60 °C and at pH 5.0–9.0, and it is not a metal dependent enzyme, which makes it robust for storage and transportation, ultimately holding great promise in green biotechnology and biorefining.

## 1. Introduction

Glycosidase, also known as glycoside hydrolase (GH), refers to a class of enzymes that have the ability to hydrolyze the glycosidic bonds between two or more carbohydrates and between carbohydrates and non-carbohydrates [1]. It plays an indispensable role in the hydrolysis and remodeling of sugars and glycoside compounds in animals, plants and microorganisms. Glycosidases can be divided into endoglycosidases and exoglycosidases according to the different cutting methods of oligosaccharides and glycoside substrates [2]. The main action sites of endoglycosidase are oligosaccharides and the internal glycosidic bonds of polysaccharides, which are mostly used to cut polysaccharides from glycoproteins such as endo- β- N-acetylglucosaminidase (EC3.2.1.96) and can cleave the bond between two N-acetylglucosamine (Glc NAc) subunits that are directly close to the asparagine residue of glycoprotein. Mitsudome et al. (2014) found that recombinant endo-β-N-acetylglucosaminidase purified from silkworm lymph can deglycosylate high-mannose glycoprotein, which is considered as an important tool for glycobiology research [3]. Exoglycosidase mainly acts on the glycosidic bond at the non-reducing end and is mainly used to study the cleavage of specific terminal groups of free polysaccharides, such as β-galactosidase (EC3.2.1.23), which can hydrolyze the β-1,4-glycosidic bond between galactose and N-acetylglucosamine under relatively mild conditions. Among the glycoside hydrolase characterized so far, in terms of the feasibility of their application in biotechnology, the microbial glycoside hydrolase is not only stable in nature, but also can easily be efficiently expressed in low-cost expression systems, and therefore meets the requirements of large-scale application in industry. Glycoside hydrolase is widely used in food, bio-energy, feed, paper, and other industrial production [4,5]. Therefore, it has attracted the attention of relevant scholars.

β-glucan is an important polysaccharide in plants, fungi, yeast, algae, and other organisms, and has a variety of physiological functions in these organisms [6]. It is a polymer composed of glucose with different degrees of polymerization (DPs), branches and different glycosidic bonds, such as β-1,3-, β-1,4-, β- 1,6-, β-1-3/1-4- and β-1-3/1-6- [7].

β-1,3-glucan is a polymer that is polymerized by the β-1,3-glucoside bond, some natural β-1,3-glucan also contains different proportions and sizes of branched chains connected by the β-1,6-glucoside bond. For example, the side chain of laminarin in brown algae contains about 30% β-1,6-linked branched chain structure and is water soluble [8]. β-1,3-glucan has rich biological activities. It can promote the proliferation of macrophages in the liver and spleen, promote their phagocytic activity, and also have the effects of antioxidation and promoting wound healing speed [9]. In addition, because of its low heat, high water holding capacity, and good biological activity, β-1,3-glucan can also be widely used in the food industry as a functional factor, thickener, fat substitute, and dietary fiber with good application prospects [10].

The natural metabolism of β-1,3-glucan is carried out through β-1,3-glucanase. β-1,3-glucanase can degrade polysaccharides linked by β-1,3-glucoside bonds into glucose or oligosaccharides, and is widely found in fungi, bacteria, algae, mollusks, and plants, among which fungi is the main enzyme source for various practical uses [11,12]. It plays an important role in the biodegradation, reconstruction and development of β-1,3-glucan. β-1,3-glucanase has different physiological functions in different organisms. For example, in fungi, β-1,3-glucanase activity is involved in development and differentiation, cell wall biogenesis, and parasite defense and nutrition, while in plants it is mainly involved in pathogen defense, and in bacteria it is involved in the acquisition of nutrients [13,14]. β-1,3-glucanases of various origins have been reported to improve the strains used in industry. For example, beta-1,3-glucanase from *Aspergillus oryzae* expressed in *Saccharomyces cerevisiae* enables yeast to degrade cellulosic materials [15,16]. Similarly, to enhance oligoglucoside hydrolysis, a thermostable β-1,3-glucanase from *Trichoderma harzianum* was expressed in *Pichia pastoris* [11,17]. Meanwhile, β-1,3-glucanases from *Chitinophaga pinensis* hydrolyze β-1,6-glucan pustulan and can be classified into different GH families with different levels of β-1,6-glucanase activity [18]. In the control of fungal pathogens, some biocontrol agents showed interest in beta-1,3-glucanase production. For example, *Bacillus subtilis* produced beta-1,3-glucanase and exerted antifungal activity [19]. Entomopathogenic fungi such as the fungus *Beauveria bassiana Vuill* are also valuable antagonists of pathogenic fungi, as they produce a β-1,3-glucanase useful for biotechnology [20]. Anaerobic bacteria such as *Thermotoga maritima* MSB8, whose optimum temperature is 80 °C, also show interest in producing β-1,3-glucanase active at elevated temperatures [21].

According to their mechanism of action, β-1,3-glucanase is divided into exo-β-1,3-glucanase (EC 3.2.1.58) and endo-β-1,3-glucanase (EC 3.2.1.39) [22]. The exo-β-1,3-glucanase can gradually break the β-1,3-glucoside bond from the non-reducing end, releasing glucose residues to produce the hydrolyzed product glucose. Endo-β-1,3-glucanase, also known as laminarinase, is an enzyme that specifically hydrolyzes the β-1,3-glucan glycosidic bond, and then breaks the β-1,3-glucan glycosidic bond randomly in the β-1,3-glucan chain to generate oligosaccharides with low degree of polymerization and release the oligosaccharide mixture [17,23]. In addition to the preparation of oligosaccharides, β-1,3-glucanase can also be used as an additive or an amendment of bioactive polysaccharide in feed and wine industry [24]. As a tool enzyme for processing yeast cells, it has been applied in protoplasmic preparation, cell fusion, gene introduction, and protein extraction [25]. β-1,3-glucanase can effectively improve the disease resistance of plants [26], and it plays an important role in the degradation of plant biomass [27].

How fungi can produce β-1,3-glucanase, given that their cell walls are rich in β-glucan, is an intriguing question. Research on this curious fact showed that some species, such as *Penicillium sumatraense*, have more than one of the enzyme-modifying genes 1,3-β-glucan in their genome. All of these glucanases have a specific modifying function, some removing glucose from the end, and others increasing the β-glucan branching system of the fungus, making the fungus more resistant to its own hydrolysis activity. This characteristic enables the fungus to feed on glycannic nutrients without damaging the organism, thus justifying the specific interest for *P. expansum* glucanases [28].

To the best of our knowledge, research on cloning and expression of *P. expansum β*-1,3-*glucanase* gene has not been reported on. In the present study, the expression and cloning of the *β*-1,3-*glucanase* gene *PeBgl*1 of *P. expansum* has been investigated for the first time.

## 2. Materials and Methods

### 2.1. Materials and Reagents

*P. expansum* strain K1 was isolated by our research team (maintained at the China Center for Type Culture Collection and numbered as CCTCC AF 2022039) [29]. *E. coli* DH5α, the host *E. coli* Rosetta (DE3), and plasmid pET-30a are the strains and plasmids preserved in our laboratory. The restriction endonucleases XhoI and NcoI were purchased from TaKaRa Company, Beijing, China. Various reagents were purchased from Sinopharm Chemical Reagents Co., Ltd., Shanghai, China (Mainland) and 2xEs Taq MasterMix was purchased from Kangwei Century, Beijing, China and the plasmid DNA small quantity purification kit from TaKaRa, Beijing, China.

### 2.2. Preparation of Culture Medium and Reagent

Luria-Bertani (LB) liquid medium (10 g peptone, 5 g yeast extract, and 10 g sodium chloride in 1000 mL distilled water) and Luria-Bertani (LB) solid medium (15 g agar powder in 1000 mL LB liquid medium) were prepared. In addition, Kanamycin (Kana) (0.5 g kanamycin dissolved in 10 mL distilled water) was prepared as a stock solution with a concentration of 50 mg/mL.

IPTG was prepared by weighing 2.4 g isopropyl- β- D-thiogalactoside (IPTG) and dissolving it in 10 mL of distilled water and then preparing it in 1 M stock solution. The laminarin solution was prepared by using a 5 mmol/L Na_2_HPO_4_-NaH_2_PO_4_ buffer solution with a pH of 7.0 and a final concentration of 4 mg/mL.

### 2.3. Construction and Transformation of Recombinant Plasmid pET-30a-PeBgl

The primers were designed using the Primer Premier 5.0 software, which was synthesized by Sangon Biotech (Shanghai, China) Co., Ltd., and the NcoI and XhoI restriction enzyme sites were added to the upstream and downstream primers of *PeBgl*1 gene, respectively. The *PeBgl*1 sequences containing enzyme restriction sites were amplified by EX-F (5′CGACCATGGTCTCTTTCACCAAGCTTTTC3′) and EX-R (5′CGACTCGAGCGCAGAGACGTAAGCTTG3′) primers. The ligation system contained 1 μL pMD18-T vector (50 ng/µL), 5 μL Solution I and 4 μL (0.1 pmol–0.3 pmol) *PeBgl*1 gene fragment. After overnight ligation at 4 °C, the ligation product was transformed into *E. coli* DH5α competent cells. The positive clones were identified by PCR (T100, BIO RA D Bole) using PeBgl1-138F (5′ATTCTGGGATTCAACGAGC3′) and PeBgl1-138R (5′GGTGGAGGAGGTTACGG3′) primers. The positive clones identified were inoculated in 5 mL LB liquid medium containing ampicillin (100 μg/mL) and cultured overnight at 180 rpm at 37 °C.

The pMD18-T-PeBgl1 plasmid was extracted according to the TaKaRa plasmid DNA small quantity purification kit. Plasmids pMD18-T-PeBgl1 and pET-30a were digested by NcoI and XhoI restriction enzymes (37 °C for 2 h). The digestion products were subjected to agarose gel electrophoresis (Beijing Liuyi Instrument Factory) to recover the target fragments PeBgl1- and pET-30a- after digestion. Then, the fragments were connected by T4 DNA Ligase. pET-30a-PeBgl1 was transformed into *E. coli* Rosetta (DE3) competent cells and coated with kana (100 μg/mL). Positive clones were identified by PCR and sequenced. Then, the *E. coli* Rosetta (DE3) containing pET-30a-PeBgl1 were obtained.

### 2.4. Expression and Purification of PeBgl1 Protein

Monoclonal pET-30a-Rosetta and pET-30a-PeBgl1-Rosetta grown on the LB plate and inoculate were taken into a 5 mL (containing 50 μg/mL Kana) liquid medium, 37 °C, 180 rpm oscillation culture for 12–16 h. Then, they were transferred into 50 mL LB liquid medium (containing 50 μg/mL Kana) at 37 °C and cultured at 180 rpm for 3–4 h until the OD value reached 0.6–0.8. An appropriate amount of IPTG with a concentration of 1 M was added to make the final concentration of IPTG in the medium 0.2, 0.4, 0.6, 0.8, and 1.0 mM, and incubated at 16 °C for 20 h to determine the final concentration. After the induced expression, the bacteria were collected and the recombinant protein was obtained, and the induced bacterial solution was taken for SDS-PAGE. The samples were used in AKTA Pure protein purification apparatus for ion exchange chromatography. Then, 5 μL of the protein samples were taken for SDS-PAGE verification. The concentration of purified enzyme liquid protein was determined according to Bradford’s Coomassie brilliant blue method [30], and bovine serum albumin (BSA) was used as the standard substance.

### 2.5. Determination of PeBgl1 Enzyme Activity

The method of DNS was used to determine PeBgl1 activity [31]. We added 50 μL laminarin solution (4 mg/mL) to 150 μL enzyme solution, and the reaction was performed at 55 °C for 30 min. After that, 300 μL DNS reagent was added and the reaction was terminated in a boiling water bath for 5 min. The absorption value of the reaction solution was measured at 540 nm in the inactivated enzyme solution with substrate solution as the control. The content of the reducing sugar was determined by making a standard curve of glucose, which was used to calculate enzyme activity. The enzyme activity of laminarin was defined as the amount of the enzyme required to produce 1μmol of reducing sugar (measured in glucose) per minute from the hydrolyzed substrate is 1 unit of enzyme activity (U).

### 2.6. Study of the Enzymatic Characteristics of PeBgl1

The enzymatic characteristics of PeBgl1 protein were studied according to the following method.

#### 2.6.1. Substrate Specificity of PeBgl1

The substrate solutions of laminarin, laminaribiose, laminarihexaose, sodium carboxymethyl cellulose, gentiobiose, maltose, salicin, xylo-oligosaccharide, and chitin were prepared. Then, the enzyme activity of each group was determined according to the enzyme activity determination method in Section 2.5.

#### 2.6.2. Optimum Reaction Temperature and pH of PeBgl1

In order to determine the optimum reaction temperature of PeBgl1, 10 groups of reaction temperatures were set. The groups were 20, 25, 30, 35, 40, 45, 50, 55, 60, and 70 °C. The enzyme activity under the above conditions was determined according to the enzyme activity determination method in Section 2.5.

In order to determine the optimum reaction pH of PeBgl1, laminarin solutions with different pH levels of were prepared with a citric acid-Na_2_HPO_4_ buffer (pH 3.0–5.0), Na_2_HPO_4_-NaH_2_PO_4_ buffer (pH 6.0–8.0), and a Na_2_CO_3_-NaHCO_3_ buffer (pH 9.0–10.0). The pH of the buffer was detected by a PH meter (pHS-3C, Shanghai Yiheng Technology Co., Ltd., Shanghai, China). The enzyme activity of each group at the optimum temperature was determined according to the enzyme activity determination method in Section 2.6.

#### 2.6.3. Effect of Na_2_HPO_4_-NaH_2_PO_4_ Buffer and NaCl Buffer Concentration in Substrate Solution on the Activity of Recombinant Enzyme

Buffers containing 5, 50, 100, and 200 mmol/L Na_2_HPO_4_-NaH_2_PO_4_ and a NaCl buffer were used to prepare 4 mg/mL of laminarin solution. The enzyme activity of each group at the optimum temperature was determined according to the enzyme activity determination method in Section 2.6.

#### 2.6.4. Effects of Metal Ions and Surfactants on Recombinant Enzyme Activity

Aqueous solutions of CuSO_4_, CaCl_2_, BaCl_2_, MgSO_4,_ and ZnSO_4_ with a concentration of 50 mmol/L were prepared. Then, 1.0% SDS, TritonX-100, and Tween-20 aqueous solution were prepared. The above solution and the laminarin solution were diluted together to the same concentration until the final concentration is 5 mM. The enzyme activity of each group at the optimum temperature was determined according to the enzyme activity determination method in Section 2.6.

## 3. Results and Discussion

### 3.1. Construction of Recombinant Plasmid pET-30a-PeBgl1-Rosetta

**Figure 1:**
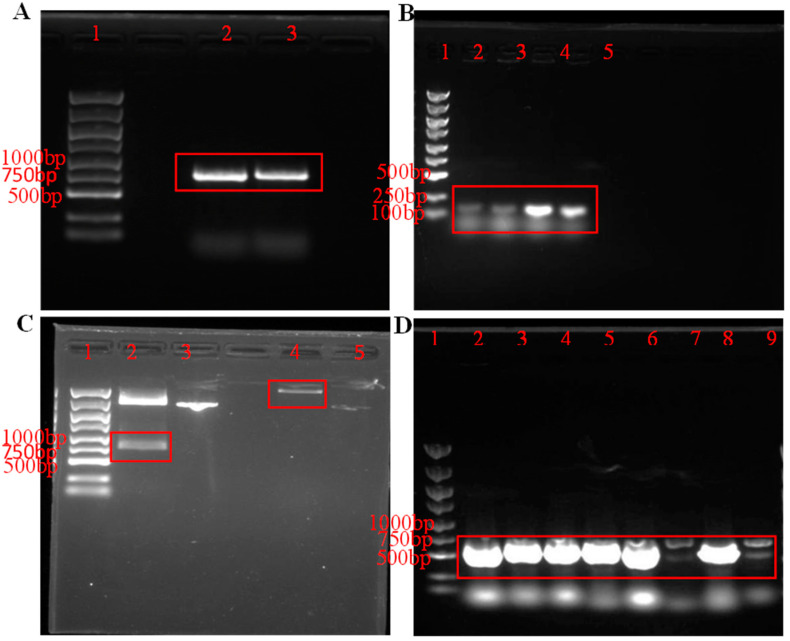
Construction of recombinant plasmid pET-30a-PeBgl1-Rosetta. (**A**) *PeBgl1* was amplified by PCR. (**B**) The *E. coli* DH5α translocator containing pMD18-T-PeBgl1 was identified by PCR. (**C**) Double enzyme digestion of pMD18-T-PeBgl1 and pET-30a plasmid. (**D**) pET-30a-PeBgl1-Rosetta was identified by PCR. Lane 1, 5000 bp DNA ladder marker; Lane 2 to 9, PCR products; Red box represents the target bands.

As shown in Figure 1A, the band size of the PeBgl1 target product was around 800 bp, corresponding to the size of the PeBgl1 gene sequence (798 bp). PCR-verified positive transformants (Figure 1B) and the four unique colonies identified all contained the PeBgl1 target gene. Eight unique colonies were identified, and the target bands were successfully amplified for eight colonies, as shown in Figure 1D.

The advent of molecular biology makes it possible to express proteins and protein complexes using different hosts. *E. coli* has extremely fast growth kinetics and can obtain high cell density, low cost of medium and reagent, and simple transformation of expression constructs [32]. Prokaryotic expression systems can obtain a large number of gene expression products in a short time, which is simple and relatively low cost. In the process of prokaryotic protein expression, tag proteins are usually added to the N-terminal or C-terminal of the target protein to facilitate the later protein purification process. Currently, commonly used tag proteins are generally divided into protein molecules (or protein domains and their derivatives) with a large relative molecular weight and polypeptide fragments with a small relative molecular weight (His-tag), etc. His-tag protein is mainly used for the purification of recombinant proteins by affinity chromatography and is one of the widely used recombinant protein purification tags at present, but it may has some disadvantages [33,34]. There are numerous examples of His-tag proteins interfering with protein solubility, structure, and even function [35,36,37,38]. The purification method using His-tag protein and nickel column affinity may lead to the inclusion of metal ions in the system of subsequent experiments, thus generating immunogenicity [38,39]. Moreover, the specificity of the His-tag protein’s affinity with nickel column is not high, resulting in low purity in the obtained protein products [40]. The expression vector is the core of prokaryotic expression, and the selection of an appropriate expression vector is conducive to the smooth process of protein expression. At present, there are three types of expression vectors used in *E. coli*: fusion, non-fusion, and secretory expression vectors. The expression vector pET-30a used in this study belongs to the fusion expression vector, which is easy to translate and can improve the stability of mRNA transcription. Importantly, it has His-tags and the subsequent purification of protein is simple and easy, which is conducive to the smooth progress of the experiment [41]. Currently, pET-30a is a highly efficient prokaryotic expression vector widely used [42]. There are two His-tags before and after the MCS region of the plasmid skeleton, and the His-tag protein can be expressed in the N-terminal or C-terminal of the fusion protein according to the reading frame. Furthermore, it has the Lac/Pt7Lac promoter, which can express a large amount of protein in the presence of inducers. pET expression vectors usually lead to inclusion bodies containing recombinant proteins, but it is rare that recombinant proteins obtained using pET vectors are not overexpressed. Yang Shikun et al. [43] successfully constructed the prokaryotic expression system of human polyamine regulatory factor-1 (PMF-1) by using pET-30a as the expression vector, and obtained the target protein with high purity and concentration by using Ni-DNA affinity column, which laid a solid foundation for the preparation of antibodies.

### 3.2. Expression and Purification of PeBgl1 Protein

**Figure 2:**
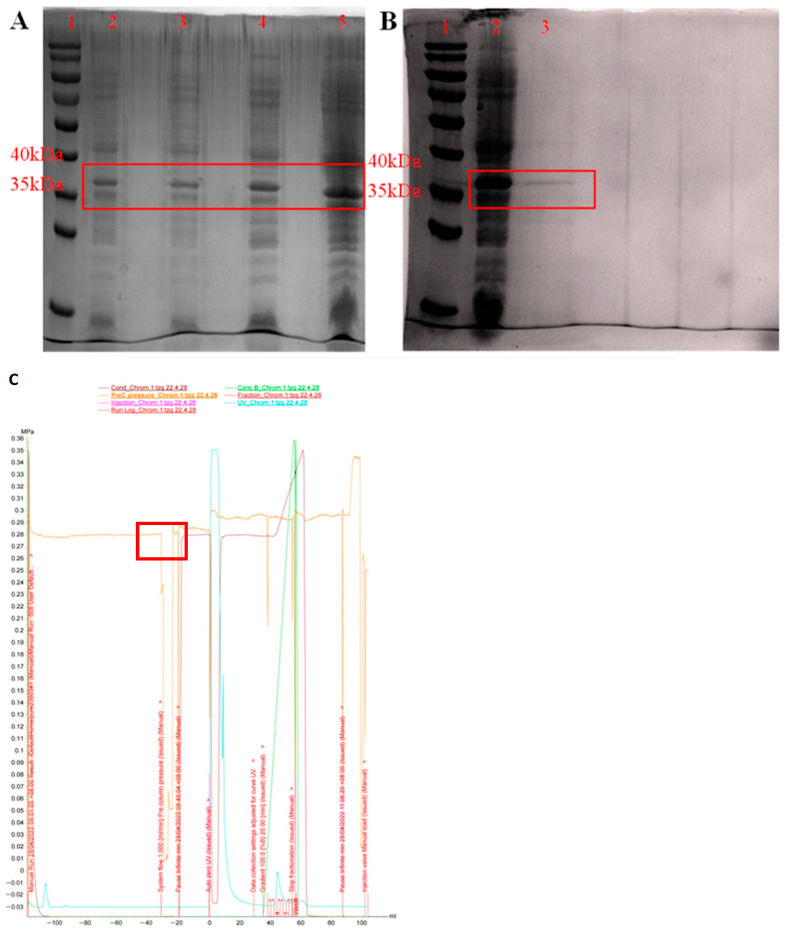
Induced expression of the recombinant PeBgl1 protein. (**A**): SDS-PAGE analysis of the recombinant induced products. Lane 1, molecular mass standard; Lane 2 and 3, protein bands from the pET-30a-Rosetta and pET-30a-PeBgl1-Rosetta at 16 °C for 20 h; Lane 4 and 5, protein bands from the pET-30a-Rosetta and pET-30a-PeBgl1-Rosetta induced by 0.8 mmol/L IPTG at 16 °C for 20 h; Red box is the target bands. (**B**): SDS-PAGE analysis of purified recombinant induced products. Lane 1, molecular mass standard; Lane 2, unpurified protein bands from the pET-30a-PeBgl1-Rosetta; Lane 3, purified protein bands from the pET-30a-PeBgl1-Rosetta; Red box is the target bands. (**C**): Protein ion exchange chromatography. The red box in Figure 2C is the interval for collecting samples.

After the induced expression by IPTG, the collected intracellular cleavage supernatant of *E. coli* was added with an appropriate loading buffer, and the un-induced *E. coli* was used as the control for SDS-PAGE analysis. The results are shown in Figure 2A, the recombinant vector showed obvious protein expression compared to the control group under the induction of 0.8 mM IPTG at 16 °C. The results showed that the recombinant protein was successfully heterologously expressed in the expression system of *E.coli*. However, *E. coli* Rosetta, as a pathogenic bacterium, contains endotoxin (lipopolysaccharide), and easily forms inclusion bodies [44]. As an expression host, it has its limitations, as some protein can be lost in precipitation.

With the UNICORN 7.1 software, the AKTA Pure protein purifier was utilized to set up corresponding procedures for the ion exchange chromatography of protein samples to remove the foreign proteins with large charge differences. The ion exchange chromatography process is shown in Figure 2C, and the results were in line with expectations. The samples were verified by the SDS-PAGE after gel filtration chromatography. Thus, relatively pure PeBgl1 protein was obtained, and the protein concentration was 3.5 mg/mL determined by BCA kit.

Currently, many *glycoside hydrolase* genes derived from animals, plants, fungi, bacteria, and archaea have been cloned and expressed. The cloning and expression of these *glycoside hydrolase* genes are basically made use of the prokaryotic expression system of *E. coli* and the eukaryotic expression system of *Pichia pastoris*. For example, researchers cloned a new β-glucosidase of the GH5 family from the genome of *Thermobifida halotolerans* and heterologous expressed it in *E. coli* BL21 (DE3). A bioactive protein product with a molecular weight of 49.6 kDa was obtained [45].

### 3.3. Study of PeBgl1 Substrate Specificity

Through the reaction with polysaccharides substrates with different bonding modes or different monomers, the characteristics of PeBgl1 hydrolytic substrate were investigated (Table 1). PeBgl1 acts primarily on substrates that contain β-1, 3-glucoside bonds. Under standard conditions, the activity of this enzyme was the highest on the substrate laminaribiose containing only β-1, 3-glucoside bonds, but it was relatively low on the substrate laminarin containing both β-1, 3-glucoside bonds and β-1, 6-glucoside bonds (mostly β-1, 3-glucoside bonds). The results were similar to those of β-1,3-glucanase derived from *Bacillus xylophilus* and *Agaricus brasiliensis* [46,47].

### 3.4. Optimum Reaction Temperature and pH of PeBgl1

**Figure 3:**
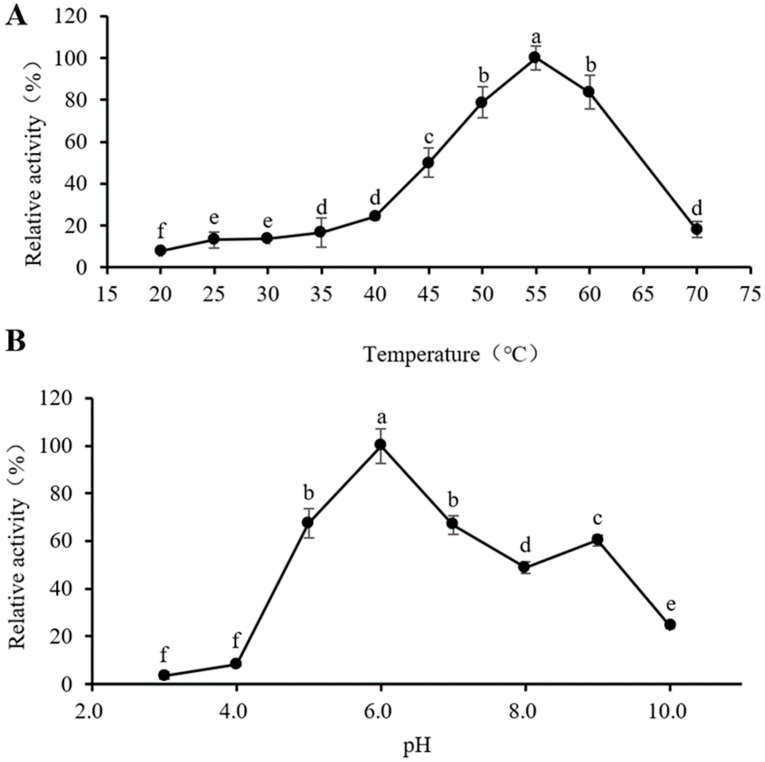
Optimum reaction temperature and pH of PeBgl1. (**A**) The optimum reaction temperature. (**B**) Optimum reaction pH. Symbols (means ± S.E.). The different letters above a, b, c, d, e and f indicate significant differences among the data. Duncan’s multiple range test method was used to analyze samples (*p* < 0.05).

With the increase in temperature, the enzyme activity of PeBgl1 gradually increased, and the enzyme activity began to decrease after exceeding 55 °C (Figure 3A), indicating that the optimum reaction temperature of PeBgl1 was 55 °C. When the treatment temperature was 70 °C, the enzyme activity was almost lost, probably due to thermal denaturation of the enzyme, resulting in the loss of the active three-dimensional structure and, hence, the loss of functionality of the enzyme’s active site [48]. Other endo-β- 1,3-glucanase also showed the highest activity at about 50 °C [49,50].

In the range of pH 3.0–6.0, with the increase in pH, the enzyme activity of PeBgl1 gradually increased. After pH exceeds 6.0, the enzyme activity gradually decreases with the increase in pH (Figure 3B), indicating that the optimal reaction pH of recombinant PeBgl1 is 6.0. PeBgl1 had the highest enzyme activity at pH 6.0, which is consistent with β-1,3(4) -glucanase (Lic16A) of *Clostridium thermocellum* [51]. However, the optimum pH of PeBgl1 was higher than that of *Humicola insolens* Y1 [52]. When the pH is too high or too low, the enzyme activity decreases rapidly. It is plausible that extreme pH values in the reaction medium destabilize the overall structure of the enzyme, or affect the combination of the enzyme and the substrate, leading to a reduction or total loss of enzymatic activity [53]. The specific mechanisms of pH effect needs further study.

### 3.5. Effect of Buffer Concentration in Substrate Solution on Enzyme Activity

**Figure 4:**
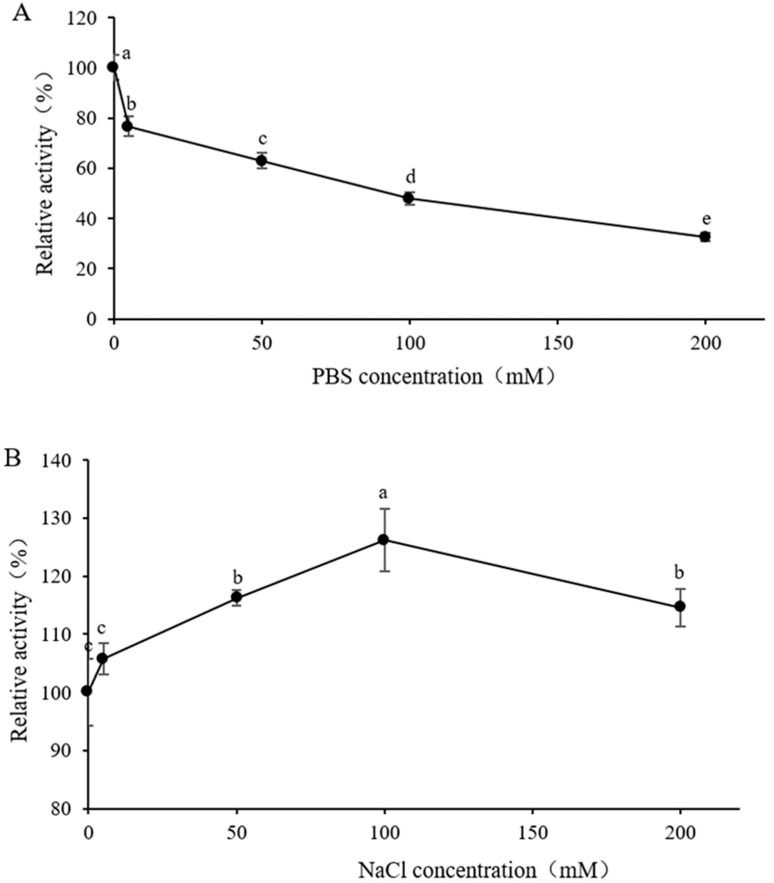
Effect of buffer concentration in substrate solution on enzyme activity. (**A**) Na_2_HPO_4_-NaH_2_PO_4_ buffer; (**B**) NaCl. Symbols (means ± S.E.). The different letters above a, b, c, d and e indicate significant differences among the data. Duncan’s multiple range test method was used to analyze samples (*p* < 0.05).

It can be seen from Figure 4A that using Na_2_HPO_4_-NaH_2_PO_4_ buffer solution with pH 6.0 to prepare 4 mg/mL of laminarin substrate solution has certain inhibition effect on enzyme activity. When the Na_2_HPO_4_-NaH_2_PO_4_ buffer was added, the enzyme activity gradually decreased with the increase in concentration. When the concentration of the Na_2_HPO_4_-NaH_2_PO_4_ buffer reached 200 mmol/L, the enzyme activity decreased below 60%. Enzyme activity peaked when 100 mmol/L NaCl was added to the substrate solution of laminarin.

### 3.6. Effect of Metal Ions and Surfactants on Enzyme Activity

**Figure 5:**
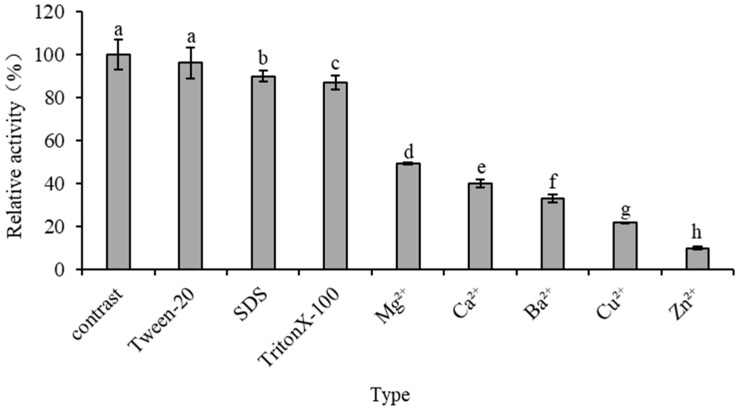
Effect of metal ions and surfactants on the enzyme activity of PeBgl1. Symbols (means ± S.E., *n* = 3). The different letters above, a, b, c, d, e, f, g and h, indicate significant differences among the data, and the Duncan method was used to analyze samples (*p* < 0.05).

It can be seen from Figure 5 that the activity of PeBgl1 was significantly affected by some metal ions and chemical reagents. Specifically, some surfactants did not have a significant impact on the activity of the recombinant enzyme. The presence of Zn^2+^ significantly inhibited the activity of the recombinant enzyme, and only 5 mM reduced the activity of the recombinant enzyme by 90%. It has been reported that low concentrations of Ca^2+^ can improve the efficiency of polysaccharide degradation by glycoside hydrolase. For example, Krishnan et al. (2019) used β-1,3-glucanase LPHase of the GH64 family as a research object and found that Ca^2+^ doubled the enzyme activity of LPHase [54]. However, Mg^2+^, Ca^2+^, Ba^2+^, and Cu^2+^ had different degrees of inhibition on the activity of the recombinant enzyme PeBgl1, which may be due to the interference of these ions with some of the key amino acids making up the PeBgl1 protein. Alternatively, it could change the spatial structure of the enzyme or bind to the functional group of the protein and cause the enzyme activity to be inhibited. These differences may be related to factors such as the glycosylation of the amino acids of recombinase, the conformation or amino acid ionization of recombinase in the reaction system, and the binding of the recombinase active site and substrate [55]. TritonX-100 and Tween 20 have inhibitory effects on enzyme activity, indicating that hydrophobic regions may play a role in the catalytic mechanism of PeBgl1. As SDS is well known to alter the spatial conformation of proteins and enzymes, the inhibition by SDS observed in this study suggests that PeBgl1 requires its characteristic three-dimensional shape to cleave its glycosylated substrates. Overall, the recombinant enzyme seems highly sensitive to metal ions and chemical reagents.

## 4. Conclusions

β-Glucan defined as β-D-glucose polysaccharide is found in plants, fungi, algae, and other organisms. It is a valuable bioresource for health, bioenergy, biomaterials, or the food industry. β-1,3-glucanases are a key class of enzymes that catalyze the hydrolysis of the glycosidic bonds of β-glucan, reducing its molecular weight and thereby modifying its physicochemical properties. As various organisms produce β-1,3-glucanases to meet specific needs, their value depends on the organism of origin, yet the *P. expansum* glucanase, PeBgl1, has so far been poorly reported. In this study, an endo-β-1,3-glucanase from *P. expansum* was expressed, purified and characterized in *E. coli* Rosetta (DE3) for the first time. The activity shown by PeBgl1 was significant and effective in hydrolyzing laminarin comparable to existing endo-β-1,3-glucanases. PeBgl1 exhibits an optimal reaction temperature and pH of 55 °C and 6.0, respectively. The PeBgl1 protein has stable enzymatic activity at 50–60 °C and pH 5.0–9.0 and is not a metal-dependent enzyme. The present study is instrumental in providing a theoretical basis for the future exploration and applications of PeBgl1. Ultimately, further research into recombinant endo-β-1,3-glucanase will contribute to broadening the field of biotechnology of high molecular weight polysaccharides, such as laminarin, scleroglucan, curdlan, and schizophyllan, to generate new biologically active compounds.

## Figures and Tables

**Table 1:** PeBgl1 activity on different polysaccharide.

Substrates	Major Glycoside Linkage (s)	Relative Activity (%)
Laminarin	1,3-β	32.11 ^c^
Laminaribiose	1,3-β	100 ^a^
Laminarihexaose	1,3-β	64.74 ^b^
CMC-Na	1,4-β	0.52 ^d^
Gentiobiose	1,6-β	0
Maltose	1,4-α	0
Salicin	D-glucose	0.66 ^d^
Xylo-oligosaccharide	1,4-β	0
Chitin	1,4-β	1.83 ^d^

The different letters (^a^, ^b^, ^c^ and ^d^) indicate significant differences. The Duncan method was used to analyze samples (*p* < 0.05).

## Data Availability

The datasets generated during and/or analysed during the current study are available from the corresponding author on reasonable request.
